# Research on the doctors’ win in crowdsourcing competitions: perspectives on service content and competitive environment

**DOI:** 10.1186/s12911-023-02309-x

**Published:** 2023-10-05

**Authors:** Xiuxiu Zhou, Shanshan Guo, Hong Wu

**Affiliations:** 1 Department of Psychiatry, Wuhan Mental Health Center, Wuhan, China; 2 School of Medicine and Health Management, Tongji Medical College, Huazhong University of Science and Technology, Wuhan, 430030 Hubei China

**Keywords:** Crowdsourcing competition, Medical crowdsourcing, Doctors’ wins, Competitive environment, Online healthcare service

## Abstract

Medical crowdsourcing competitions can help patients get more efficient and comprehensive treatment advice than “one-to-one” service, and doctors should be encouraged to actively participate. In the crowdsourcing competitions, winning the crowdsourcing competition is the driving force for doctors to continue to participate in the service. Therefore, how to improve the winning probability needs to be revealed. From the service content and competitive environment perspectives, this study introduces doctor competence indicators to investigate the key influence factors of doctors’ wins on the online platform. The results show that the emotional interaction in doctors’ service content positively influences doctors’ wins. However, the influence of information interaction presents heterogeneity. Conclusive information helps doctors win, while suggestive information negatively affects them. For the competitive environment, the competitive environment negatively moderates the relationship between doctors’ service content and doctors’ wins. The results of this study provide important contributions to the research on crowdsourcing competitions and online healthcare services and guide the participants of the competition, including patients, doctors, and platforms.

## Introduction

Due to its features such as convenience, anonymity, and comprehensive functions, online healthcare service has gradually become an important supplement to traditional medical services, and patients choose to consult online to obtain professional medical suggestions and health services [1, 2]. However, with the continuous development of patients’ health needs, the current mainstream service of “one-to-one” is not enough to meet the diverse needs of patients. On the one hand, limited by time and resources, doctors’ increased offline workload may reduce their availability to provide online services [3], under the “one-to-one” service, patients often need to wait a long time, and their needs often cannot be met promptly. On the other hand, service from one doctor sometimes cannot be sufficiently trusted or satisfied by the patient. Especially for complex diseases, patients often consult multiple doctors successively and combine the responses of multiple doctors and mutual verification to obtain a definitive diagnosis.

The emergence of crowdsourcing services provides a possibility to improve the above limitation of “one-to-one” service. In recent years, medical crowdsourcing service has emerged in health institutions and has shown strong application advantages [4]. Under the crowdsourcing service, doctors can learn from each other, communicate, and share experiences that are conducive to finding a breakthrough point to solve diseases in a short time [5]. Patients can also obtain more medical information and treatment suggestions to alleviate the information asymmetry between doctors and patients, thus reducing the information uncertainty of patients and improving their medical experience.

Based on the crowdsourcing model, new opportunities and developments have emerged in the online medical community. Different from the “one-to-one” online consultation service, the crowdsourcing service can gather multiple doctors in a short time to ensure that users can get answers in the fastest time when they ask for suggestions at any time [4]. The medical crowdsourcing service has been adopted in online healthcare platforms and is widely used including Guahao.com and 39Health.com.

Existing studies on online healthcare services mainly focus on the “one-to-one” service, and investigate doctor-patient behaviors [3, 6, 7], doctor participation results [8], patient participation results [9, 10] and spillover effects of online participation [11, 12]. However, few studies focus on the medical crowdsourcing service. Research on crowdsourcing mode mainly focuses on its application in the field of public health, and discusses its application in the field of preventive medicine [13], public health [13, 14], health information communication and health education [15], as well as in the field of biology and epidemiology [16–18], emphasizing the practical value of crowdsourcing collaboration. There are few in-depth studies from the perspective of competition.

As the service provider, the online healthcare platform provides a typical competitive environment for doctors. Based on the social exchange theory, individuals will measure their costs and expected benefits in the process of social exchange [19]. Especially in a competitive environment, they will be willing to participate in the fierce competition when their expected benefits exceed the cost [20]. Winning in crowdsourcing services is an incentive for doctors to continue to participate in the service, and they are eager to stand out from the competition to receive a certain amount of payment or reward. Their activity decreased when their suggestions were not successfully adopted [21]. Therefore, the winning mechanism of doctors in the medical crowdsourcing competition needs to be revealed.

Thus, driven by the practical needs and theoretical gaps, this study aims to investigate the following question: ***how can doctors improve their chances of winning crowdsourcing competitions in the online environment***? In order to explore this important issue, this study selects Guahao.com, an online healthcare platform that provides crowdsourcing services in China, and obtains the crowdsourcing service data of the platform from June to December 2020. The key factors affecting doctors’ winning in the crowdsourcing competitions have been discussed by obtaining and analyzing the information on patient-doctor interactions.

## Theoretical background and hypotheses development

### Crowdsourcing competition and online healthcare service

Crowdsourcing refers to an online and distributed working mode in which a large task is divided into many small tasks through the Internet platform and completed by multiple users [22]. As it aims to solve problems by aggregating collective wisdom, crowdsourcing can not only help the contractor achieve cost reduction and efficiency, but also promote task innovation with the help of diverse expertise [23, 24]. According to the differences in working modes, crowdsourcing can be divided into two types: collaborative crowdsourcing (the task is completed through the collaboration of peers) and competition-based crowdsourcing (the task is completed only by an individual who works alone and has sufficient independence) [25, 26]. The competition-based crowdsourcing, also called crowdsourcing competition, is defined as an invitation from a private or public organizer to the public or a target group to submit a solution to a challenge within a certain permitted period [27, 28]. The initiator of the competition will set certain rewards in advance to attract participants to provide task schemes, while the participants will strive to win the competition rewards as competitors.

Medical crowdsourcing service is the application of crowdsourcing competition in healthcare. In the online environment, crowdsourcing service also has a competitive nature, which is a special kind of crowdsourcing competition. Different from “one-to-one” services, crowdsourcing competition helps to “select the best”. Patients are transformed from passive service receivers into active initiators of crowdsourcing services and have the absolute initiative in determining the winners of crowdsourcing. In general, all participants (doctors) have the knowledge or ability to solve a given task independently, doctors need to use certain strategies to stand out among the many participants as a service provider.

**Fig. 1:**
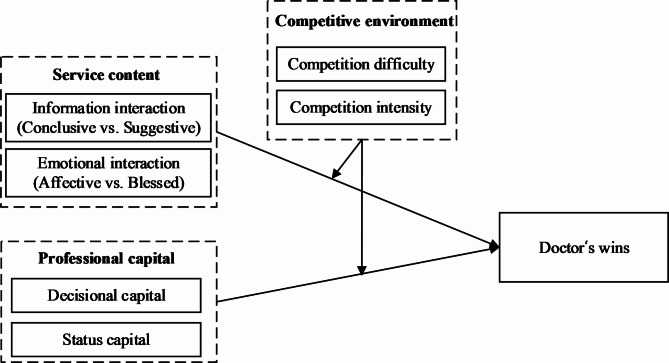
Conceptual model

Existing studies on crowdsourcing competition have extensively discussed the influence of tasks and participants’ characteristics on the results of crowdsourcing tasks, and can be divided into two main research directions. One is to explore the internal factors that affect the competition results, such as task rewards [29, 30], task difficulty [31] and task completion quality [24]. The second is to study the external environment that affects the competition results, such as the winning rate of participants [31], the number of participants [32], and the ability of participants [29]. However, few studies take both internal and external factors into consideration. Taking online healthcare platforms as the research background, this study will explore the impact of doctors’ service content (information interaction and emotional interaction) and professional capital (status capital and decisional capital) on doctors’ wins. Moreover, the moderating effects of the competitive environment (competition difficulty and competition intensity) is also introduced to investigate the influence mechanism of the competitive environment on competition results. Figure 1 shows the conceptual model.

### Service content and doctors’ wins

Most existing studies divide service content into two dimensions: information interaction and emotional interaction. In marketing, enterprises’ online information and emotional services have significant positive impacts on customer behavior [26]. In healthcare, the emotional support of members (other chronic patients in the community) has a significant positive effect on the willingness to continuous participation intention based on social support theory [33]. As a provider of online healthcare services, doctors’ social support (i.e., doctors’ information and emotional support) can significantly affect patient satisfaction (i.e., medical quality satisfaction and service attitude satisfaction) and effectively improve patients’ medical experience [34]. The rewards allocation is affected by the support and suggestions received by patients. This study divides information interaction into conclusive information and suggestive information, and divides emotional interaction into affective support and blessed support.

The usefulness of the information in the content of the doctor’s response is the main reflection of the quality of the service provided, and also the important basis for the patients to select the winners. Based on the perceived value theory, useful information can improve patient satisfaction by helping them reduce uncertainty in decision-making [35]. During the treatment process, doctors will provide patients with professional information on disease knowledge and treatment suggestions [36], including the severity of the disease, treatment options, and postoperative precautions. This clear and conclusive information can help patients eliminate panic and anxiety about the disease and their health status, relieve the existing information asymmetry between doctors and patients, and thus improve patient satisfaction and even give doctors a financial reward. It should be noted that many doctors sometimes dare not give a clear diagnostic conclusion to avoid misdiagnosis, but provide advice, such as suggesting patients to go to offline hospitals for examination and treatment in the online environment [9]. Therefore, for patients expecting clear consultation results, the above suggestive information will affect their judgment on the perceived usefulness of the service content, and thus they are reluctant to give a positive evaluation, which is not conducive to the success of doctors in crowdsourcing services. Thus, we propose the following hypotheses:

H1a: Conclusive information in doctors’ service content has a positive effect on doctors’ wins.

H1b: Suggestive information in doctors’ service content has a negative effect on doctors’ wins.

Empathy is the best bond for doctor-patient interaction and effective communication, and an important means to establish a trust relationship between doctors and patients [37]. Doctors can provide affective or blessed information to patients through emotional interaction, which is an important embodiment of personal empathy. Based on existing studies, compared with information support, emotional expression can alleviate patients’ negative emotions more directly and establish a certain doctor-patient trust relationship at the initial stage [38]. As a special kind of emotional interaction, blessed messages can release stronger signals of goodwill and help build a stronger bond of trust between individuals [39].

By expressing blessed information and passing goodwill to patients, doctors can help patients reduce their psychological pressure and actively participate in follow-up treatment [40]. The effective establishment of doctor-patient trust can greatly improve patients’ impressions and evaluations of doctors [34]. Therefore, we propose the following hypotheses:

H1c: Affective information in doctors’ service content has a positive effect on doctors’ wins.

H1d: Blessed information in doctors’ service content has a positive effect on doctors’ wins.

### Professional capital and doctors’ wins

Professional capital refers to a kind of high-quality social resources belonging to social professionals (such as teachers, doctors and lawyers), which reflects an individual’s status in social structure and influences decision-making behavior [41, 42]. Professional capital can be classified as decisional capital and status capital. Status capital is a kind of structural power certified by the official, which measures individual advantage in a social structure [6, 43]. Decisional capital is manifested through dynamic interactions of social professionals, as their interaction behavior is ensured by their capability to act independently and is secured by a code of commitment [44]. In online healthcare platforms, the status capital of a doctor is determined by a series of objective factors including the doctor’s education, academic title (e.g., professor), clinical title (e.g., chief doctor) and the ranking of the hospital, whereas the decisional capital is measured by doctors’ online behavior reflected in their interactions with patients including consultations, online working experience and patient feedbacks.

In the online environment, professional capital reflects the resources and abilities of doctors and has an important effect on patient feedback and decision-making [6, 45]. In general, doctors with higher titles and working in higher the level of hospital have more resources, and patients also believe that doctors with higher status capital will provide more reliable and valuable services and are more inclined to give positive evaluations [46]. For doctors with higher decisional capital, they have more work experience. Meanwhile, frequent online interaction can also reflect their work enthusiasm, which constitutes the dynamic online reputation of doctors. Doctor reputation not only accumulates gradually in the long-term interaction, but even forms a prior impression at the early stage of the interaction, thus making it easier for patients to adopt the advice given by doctors [46].

Considering the similarities between “one-to-one” service and crowdsourcing service in terms of service scope, service goal and service object, we believe that there may be a similar mechanism of action in medical crowdsourcing competition. Thus, we propose the following hypotheses:

H2a: The decisional capital of doctors has a positive effect on doctors’ wins.

H2b: The status capital of doctors has a positive effect on doctors’ wins.

### The moderating effects of the competitive environment

In crowdsourcing competitions, doctors who have posted responses to the same question from a patient will be competitors as the patient will only select one response that meets their own medical needs after receiving many responses. Therefore, the performance of these competitors will influence competition results except the doctor’s own performance. Higher competition difficulty means patients have encountered more doctors with the high title, hospital rating, and reputation. It is difficult to highlight the personal advantages among competitors, and professional capital, which can be used as the standard of high-quality evaluation, will weaken its signal value. The quality gap between different doctors’ service content will be narrowed accordingly, and the types of information (information interaction and emotional interaction) contained in it will also be similar. In other words, when the overall quality of doctors participating in the same question is higher, the more difficult the competition is, and the impacts of doctors’ professional capital and service content on the doctors’ wins will be weakened:

H3a: Competition difficulty has a negative moderating effect on the relationship between professional capital (decisional capital and status capital) and doctors’ wins.

H3b: Competition difficulty has a negative moderating effect on the relationship between information interaction (conclusive information and suggestive information) and doctors’ wins.

H3c: Competition difficulty has a negative moderating effect on the relationship between emotional interaction (affective information and blessed information) and doctors’ wins.

In addition, the increase in the number of competitors means that the uncertainty of the outcome of the competition will also increase.

When a large number of solvers enter the competition, screening and evaluating too many solution proposals can be challenging and costly for seekers, and even worthy solutions may not receive the attention they deserve [47, 48]. As the crowdsourcing process progresses, the more competitors participate, the more dynamic the competition becomes [49]. Existing studies find that borrowing ideas from others in crowdsourcing helped participants come up with ideas [50]. Latecomers can refer to the submitted answers to improve their service content, which means that the content gap between competitors is dynamically narrowed. Therefore, the more participants, the more competitive the crowdsourcing service.

Based on the signal theory, the competitive environment can be regarded as a signaling environment in which the characteristics and behaviors of the participants send many signals [51]. When the number of competitors increases, the number of signals participating in the competition will decrease the signal-to-noise ratio of effective signals, thus reducing the value of signals in the competitive environment and making it difficult for the initiator to evaluate the quality of the task effectively [52]. In the medical crowdsourcing service, the quality of doctors’ response content (i.e., service content) is the key for patients to judge whether the treatment or suggestion needs are met, and an important signal affecting patients’ decision-making. However, the number of signals of the service content received by patients increases with the number of competitors increases. The signal value of the service content, which can be used as a high-quality evaluation index, will be reduced due to the interference of the number of signals, and the competitive advantage of individual doctors will be less easily reflected. However, the doctor’s professional capital will serve as a supplementary signal, which can help patients judge the quality of the doctor’s response more quickly and efficiently [53]. In general, patients perceive doctors with higher professional titles and extensive experience as providing better service quality and are more likely to choose such doctors [54]. Therefore, when the number of competitors increases, the influence of doctors’ professional capital and wins is enhanced, while the influence of service content on wins is weakened.

H3d: Competition intensity has a positive moderating effect on the relationship between professional capital (decisional capital and status capital) and doctors’ wins.

H3e: Competition intensity has a negative moderating effect on the relationship between information interaction (conclusive information and suggestive information) and doctors’ wins.

H3f: Competition intensity has a negative moderating effect on the relationship between emotional interaction (affective information and blessed information) and doctors’ wins.

## Methods

### Research context and data collection

**Fig. 2:**
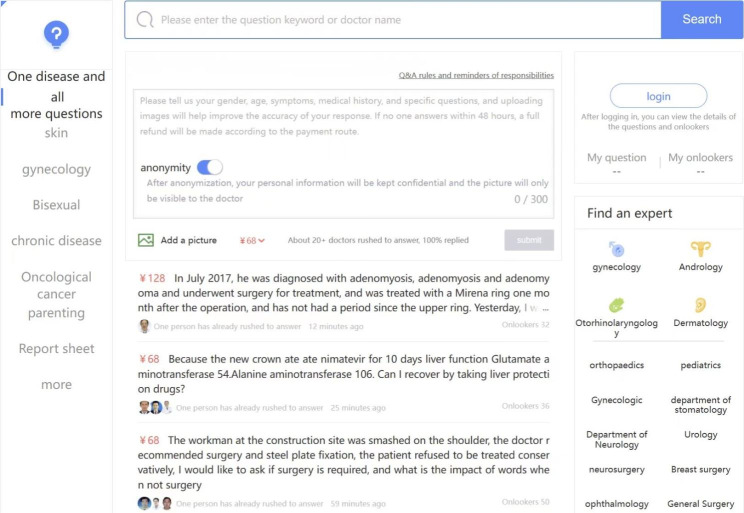
Crowdsourcing service on Guahao.com

This study collects data from Guahao.com, one of the leading online healthcare platforms in China. By May 2023, Guahao.com has more than 172,909 registered doctors, contributing to a total of about 222 million online services. This study focuses on the crowdsourcing service on Guahao.com (see Fig. 2). Different from the “one-to-one” service, patients can obtain many responses from many doctors in a short time. The operation of this service is to raise questions, doctors answer, patients evaluate and reward. First, patients ask questions to doctors through the platform, including condition description, symptoms, diagnosis, treatment and other aspects, and pay a certain reward amount (three grades: 30, 68, 128 CNY). Then, doctors can give their professional suggestions and appropriate treatment plans to patients. These responses were then rated by the patients and marked with the logo “helpful”. As of March 2021, the rules for awarding incentives under the “One Question, Many Answers” service model of Guahao.com are as follows: Doctors whose responses are labeled as “helpful” by patients will receive 50% of the amount paid by the patient. Since the platform does not limit the number of responses that can be labeled as “helpful” for a single question, when more than one response is labeled as “helpful,“ the amount is divided equally among these doctors. Alternatively, if the patient does not mark any responses as “helpful”, the amount will be divided equally among all doctors who participated in answering the question. Finally, the platform acts as an intermediary and distributes the reward to these corresponding doctors.

In order to eliminate the differences caused by different diseases, this paper only focuses on psychological diseases as the user population of such diseases is large for the crowdsourcing service. By developing a python crawler tool, we crawled the data of the psychological department in the " One Question, Many Answers " section on March 12, 2021 on Guahao.com. At the same time, we collect the personal information of the doctors who provide the program, which is saved in the database from June to December 2020. The collection process is as follows: first, crawl the question list on the homepage of " One Question, Many Answers” to get the URLs of all questions, question text, question time, and patient IDs, and then go to the question detail page according to the URLs of all questions to get the text of the replies, time of the reply, ID of the replying doctors, and whether the question is marked as “helpful” or not. Finally, based on the ID of the replying doctors, we get personal information such as the doctor’s title, the doctor’s cumulative service volume and the number of followers. Finally, 8,313 questions and 115,822 replies were obtained for the study. By obtaining information about patients’ questions, information about participating doctors, and information about the results of the crowdsourcing competition, this study was able to explore the key influencing factors of doctors’ winning in crowdsourcing competition.

### Variable measurement

The doctor’s win is introduced as the dependent variable. A dummy variable is used to measure whether a doctor’s response is adopted.

Service content is obtained by two researchers in related fields by marking the texts. First, 1000 doctor responses were randomly selected from the sample as the initial marker sample, and the two researchers individually marked the information interaction (conclusive and suggestive) and emotional interaction (affective and blessed) involved in the text. Through comparison, 89% of the samples were marked in agreement, and by discussing the inconsistent samples, the two researchers finally reached an agreement and one of them completed the marking of the remaining responses. The main marker words of text annotation are shown in Table 1.

**Table 1:** The main marker of service content

Response type		Markers
Conclusive		“Current condition is improving”, “Good recovery from treatment”, “Not much of a problem”, “Indicators are normal”, “this condition is”, “you have no symptoms”, “two examinations do not change much”, “dizziness several possibilities, you look at it in general”, “no big too serious problems”, “overall still good” etc.
Suggestive	Medication, care, daily life	“Try not to eat…”, “You can consider the option of…” The “I should actively adjust the lifestyle”, “adjust the mentality, normal life”, “do not stay up late and tired, do not Anger”, etc.
	Doctors / Departments / Hospitals	“Neurology at the Chinese Medicine Hospital is recommended.“, “It is recommended that one should go for vascular ultrasound of the neck”, “Minimally invasive surgery may be required”, etc.
Affective		“Don’t be overly nervous”, “Relax”, “Don’t worry”, “Don’t worry at all”, “It doesn’t matter”, “Don’t be afraid”, “You are so young, your chances of recovery are very good”, etc.
Blessed		“May you recover soon”, “May your baby grow up healthy”, “Good luck with your pregnancy”, “Good luck”, “Good luck. Wish you a happy life”, “Blessings of happiness and health”, etc.

For the competitive environment, competition difficulty is measured by reward amount. Existing studies find that the higher the reward amount given by the crowdsourcing task initiator, the higher the ability of the participants attracted [33]. The size of rewards determines whether high-quality participants can be attracted [30]. More rewards will attract participants with high professional abilities and improve the overall quality of participants’ responses. Therefore, this paper uses the reward amount set in the question to measure the ability of competitors, namely the competition difficulty. The competition intensity is measured by the number of competitors involved in the same question.

For professional capital, decisional capital and status capital are included. Based on the research [6, 53], this study includes the response speed and the number of patients of doctors in “one-to-one” service as the decisional capital. The title of doctor measures status capital.

This study also includes the hospital level as a control variable. Hospital level, which is also evaluated and issued by government health departments, reflects a hospital’s functions, equipment, technology, etc. There are three levels: A, B, and C, with A being the best. As the number of level C hospitals is very small, we combined level C with level B and used one dummy variable *HLEVEL* to measure the hospital level. The specific measurements of the variables are shown in Table 2.

**Table 2:** Variable measurements

Variables	Variable description
**Dependent variable**	
Doctor win (*DW*)	Whether the doctor’s response is marked as “helpful” by the patient is indicated by the Dummy variable, with 1 being that the doctor did not win.
**Independent and moderating variables**	
Conclusive information (*CI*)	Set a Dummy variable, = 1 if the doctor’s response contains conclusive information; 0 otherwise.
Suggestive information (*SI*)	Set a Dummy variable, to take a value of 1 if the doctor’s response contains suggestive information; 0 otherwise.
Affective information (*AI*)	Set a Dummy variable to take the value of 1 if the doctor responses with an affective information; 0 otherwise.
Blessed information (*BI*)	Set a Dummy variable to take the value of 1 if the doctor responses to a statement containing a blessing; 0 otherwise.
Decisional capital (*DC*)	The total number of “one-to-one” consultations with doctors is taken as a logarithmic value.
Status capital (*Pc*, *Pa*)	Set two Dummy variables *SC* and *SA*, *SC* = 1 if the doctor title is a chief doctor or associate chief doctor; 0 otherwise. *SA* = 1 if the doctor title is attending doctor or associate attending doctor; 0 otherwise.
Competition difficulty (*ComD*)	The reward amount is used as a proxy variable, i.e., the amount paid by the patient when he or she asked the question.
Competition intensity (*ComI*)	The number of responses to the question.
**Control variables**	
Hospital level (*HL*)	Set a Dummy variable, = 1 if the doctor’s hospital is a tertiary hospital; 0 otherwise.

### Empirical model

The research model of the impacts of service content and competitive environment on doctor win is as follows:

1 $$DW={\beta _0}+{\beta _1}CI+{\beta _2}SI+{\beta _3}AI+{\beta _4}BI+{\beta _5}Pc+{\beta _6}Pa+{\beta _7}DC+{\beta _8}HL+\varepsilon$$

where β_1_-β_7_ are the focus parameters to be estimated, β_0_ is the constant term and β_8_ represent the coefficients of the control variable. HL represents the control variable. ε is the error term. The detailed description for other symbols can be found in Table 2.

2 $$\begin{gathered} DW={\beta _0}+{\beta _1}CI+{\beta _2}SI+{\beta _3}AI+{\beta _4}BI+{\beta _5}Pc+{\beta _6}Pa+{\beta _7}DC+{\beta _8}ComD+{\beta _9}ComI \hfill \\ {\text{ }}+{\beta _{10}}CI \times ComD{\text{+}}{\beta _{11}}SI \times ComD{\text{+}}{\beta _{12}}AI \times ComD+{\beta _{13}}BI \times ComD \hfill \\ {\text{ +}}{\beta _{14}}CI \times ComI{\text{+}}{\beta _{15}}SI \times ComI{\text{+}}{\beta _{16}}AI \times ComI+{\beta _{17}}BI \times ComI \hfill \\ {\text{ }}+{\beta _{18}}Pc \times ComD{\text{+}}{\beta _{19}}Pa \times ComD{\text{+}}{\beta _{20}}DC \times ComD \hfill \\ {\text{ }}+{\beta _{21}}Pc \times ComI{\text{+}}{\beta _{22}}Pa \times ComI{\text{+}}{\beta _{23}}DC \times ComI{\text{+}}{\beta _{24}}HL{\text{+}}\varepsilon \hfill \\ \end{gathered}$$

where β_1_-β_9_ represent the regression coefficients of the direct effect, β_10_-β_23_ are the regression coefficients of the moderating effect, β_24_ represent the coefficients of the control variable. β_0_ is the constant term and ε is the error term. The detailed description for other symbols can be found in Table 2.

## Results

### Descriptive statistics and correlations

The descriptive statistics of all variables, Pearson correlation coefficients between variables, and their significance are shown in Table 3. From Table 3, it is clear that the mean values of suggestive and conclusive information are 0.98 and 0.84, respectively, both close to 1, which means that most of the doctors’ responses contain suggestive and conclusive information. The mean values of affective and blessed are 0.03 and 0.05, respectively, indicating that most of the doctors’ responses contained less effective and blessed information. The mean value of the number of responses is 15.06, indicating that the number of doctor participants per patient question for the patient questions studied in this paper is approximately 15.

**Table 3:** Descriptive statistical correlation analysis

Variables	Mean	Std. Dev.	VIF	1	2	3	4	5	6	7	8	9	10
1. Doctor win	0.75	0.96											
2. Decisional capital	4.37	0.61	1.07	0.164**									
3. Conclusive information	0.84	0.36	1.17	0.046**	0.177**								
4. Suggestive information	0.98	0.13	1.05	0.008**	0.107**	-0.019**							
5. Affective information	0.03	0.17	1.01	0.021**	0.024**	0.000	-0.103**						
6. Blessed information	0.05	0.21	1.02	0.035**	0.116**	0.000	0.010**	0.028**					
7. Competition difficulty	4.52	0.38	1.45	0.014**	0.143**	0.348**	0.145**	-0.047**	0.008**				
8. Competition intensity	15.06	4.84	1.00	-0.085**	-0.021**	0.023**	-0.014**	0.014**	-0.021**	0.018**			
9. Hospital level			1.02	0.010	0.028**	0.010	0.032**	0.000	-0.030**	0.124**	-0.016**		
10. Doctor title-Pc			73.96	0.012**	0.119**	0.198**	0.060**	-0.021**	0.023**	0.458**	0.011**	0.030**	
11. Doctor title-Pa			73.40	-0.012**	-0.117**	-0.197**	-0.057**	0.021**	-0.023**	-0.451**	-0.010**	-0.026**	-0.993**

**Note**: **p < 0.01

### Empirical results

The results of the linear regressions are shown in Table 4. As shown in Table 4, in terms of direct effects, conclusive information in information interaction had a significant positive effect on doctor win (*β* = 0.249, *p* < 0.001), whereas suggestive information had a negative effect on doctor win (*β*=-0.049, *p* < 0.05); Emotional information had significant positive effects on doctor win: affective information (*β* = 0.088, *p* < 0.001), blessed information (*β* = 0.074, *p* < 0.001); Competition difficulty and competition intensity have significant negative influences on doctor win (*β*=-0.032, *p* < 0.001; *β*=-0.016, *p* < 0.001). In summary, H1a, H1b, H1c, and H1d are supported.

In terms of moderating effects, the competition difficulty had a significant negative moderating effect on the relationship between doctors’ service content and doctor win: conclusive information (*β*=-0.051, *p* < 0.05), suggestive information (*β*=-0.085, *p* < 0.05); the competition intensity had a significant negative moderating effect on the relationship between blessed information and doctor win (*β*=-0.005, *p* < 0.05). Therefore, H3b is supported, H3f is partly supported, and H3c and H3e are not supported.

In contrast, there is no significant effect between status capital and decisional capital on doctor win, nor is there a moderating effect. Therefore, hypotheses H2a, H2b, H3a, and H3d are not supported.

### Robustness check

According to the rules of reward distribution in the platform: those whose responses are marked as “helpful” by patients can get 50% of the amount given by patients. As the platform has no limit on the number of “helpful” responses, when there are multiple “helpful” responses, the amount is divided equally among these doctors. Since there is no limit to the number of responses used for each question, in order to reduce the arbitrariness of patients’ choice of results, the sample with more than half of the total number of doctors who responded to the question labeled “helpful” is removed, and the results of the robustness check are obtained as shown in Table 5. As shown in Table 5, in terms of coefficients and significance, the results of doctor response content, competitive environment, and moderating effects are the same in both regressions, indicating that the results are robust.

**Table 4:** Linear regression results

Variables	Model 1		Model 2		Model 3		Model 4	
	Coefficient	Std. Dev.	Coefficient	Std. Dev.	Coefficient	Std. Dev.	Coefficient	Std. Dev.
Hospital level	0.030	0.019	0.010	0.019	0.010	0.019	0.006	0.019
Doctor title-Pc			-0.013	0.062	0.010	0.062	0.007	0.062
Doctor title-Pa			0.014	0.063	0.022	0.062	0.018	0.062
Decisional capital			0.009	0.005	0.007	0.005	0.012	0.058
Conclusive information			0.051***	0.008	0.065***	0.008	0.232*	0.116
Suggestive information			-0.049*	0.021	-0.044*	0.021	0.314	0.220
Affective information			0.088***	0.016	0.092***	0.016	0.236	0.191
Blessed information			0.074***	0.013	0.067***	0.013	-0.173	0.170
Competition difficulty					-0.032***	0.009	0.082	0.070
Competition intensity					-0.016***	0.001	0.004	0.005
Competition difficulty × Conclusive information							-0.051*	0.027
Competition difficulty × Suggestive information							-0.085*	0.049
Competition difficulty × Affective information							-0.034	0.041
Competition difficulty × Blessed information							0.070	0.046
Competition intensity × Conclusive information							0.003	0.002
Competition intensity × Suggestive information							0.000	0.003
Competition intensity × Affective information							0.000	0.003
Competition intensity × Blessed information							-0.005*	0.003
-2log likelihood	2248.74		2171.64		2165.81		2157.93	
Nagelkerke R2	0.014		0.060		0.072		0.077	

Note: *p < 0.1, ***p < 0.01

**Table 5:** Robustness check results

Variables	Model 1		Model 2		Model 3		Model 4	
	Coefficient	Std. Dev.	Coefficient	Std. Dev.	Coefficient	Std. Dev.	Coefficient	Std. Dev.
Hospital level	0.031	0.020	0.010	0.019	0.006	0.019	0.002	0.020
Doctor title-Pc	0.081	0.067	-0.008	0.066	0.002	0.066	-0.001	0.066
Doctor title-Pa	0.051	0.067	0.017	0.066	0.013	0.066	0.009	0.066
Decisional capital			0.009	0.005	0.007	0.005	0.002	0.060
Conclusive information			0.053***	0.008	0.067***	0.008	0.355***	0.123
Suggestive information			-0.055*	0.021	-0.050*	0.022	0.176	0.231
Affective information			0.088***	0.016	0.092***	0.016	0.219	0.196
Blessed information			0.077***	0.013	0.069***	0.013	-0.075	0.177
Competition difficulty					-0.016***	0.001	0.002	0.005
Competition intensity					-0.035***	0.009	0.069	0.072
Competition difficulty × Conclusive information							-0.077***	0.028
Competition difficulty × Suggestive information							-0.058*	0.021
Competition difficulty × Affective information							-0.030	0.042
Competition difficulty × Blessed information							0.052	0.037
Competition intensity × Conclusive information							0.003	0.002
Competition intensity × Suggestive information							0.001	0.004
Competition intensity × Affective information							0.001	0.003
Competition intensity × Blessed information							-0.006*	0.003
-2log likelihood	2516.48		2429.65		2426.27		2418.98	
Nagelkerke R2	0.000		0.056		0.058		0.062	

Note: *p < 0.1, ***p < 0.01

## Discussion and implication

This study examines the effects of doctors’ service content, professional capital, competition difficulty and competition intensity of their competitive environment on doctors’ wins under the medical crowdsourcing competition, and most of the hypotheses proposed are verified by the data of the online healthcare platform “Guahao.com”. The empirical results show that, except for the suggestive information, doctors’ service content has a positive impact on their wins in the crowdsourcing competition. When the service content of doctors includes conclusive, affective, blessed information, the likelihood of winning will be improved. The findings in this paper complement the relevant research on online healthcare services, and confirm the necessity of emotional interaction support for patients from the perspective of doctors. Most importantly, the conclusions of this paper confirm the adverse effect of suggestive information on doctor win, which differs from previous studies [9]. The possible explanation is that, compared with conclusive information, the certainty of suggestive information is weaker, which may affect patients’ judgment of the usefulness of the quality of the doctor’s response.

In addition, the competitive environment (competition difficulty and competition intensity) has a certain negative moderating effect on the relationship between doctors’ service content (information interaction and emotional interaction) and doctors’ wins. Specifically, when the number of competitors increases, the positive influence of information interaction in the doctor’s service content on the doctor win will be weakened. And the competition intensity of the competitive environment has a significant negative moderating effect on the relationship between blessed information and the physician’s wins. This means that as the competition intensity of the competitive environment increases, the positive effect of blessed information on physician wins is weakened. This is because patients pay more attention to the doctor’s professional competence when they visit the clinic, and the affective information will be considered as a secondary factor. And in a highly competitive environment, patients may be more inclined to choose the winning doctor based on the quality of the information in the response, rather than making a judgment based on whether the response contains a blessed information. Meanwhile, unlike affective information, which usually contain strong emotional components and have various tendencies such as sympathy, worry, and joy, affective information can convey a single emotional tendency and intensity, and the emotional stimulation they bring is easily ignored by patients in a highly competitive environment.

Furthermore, this paper also found that the crowdsourcing service differed from “one-to-one” service in some aspects, specifically in that physicians’ professional capital did not have a significant effect on physician winning in the medical crowdsourcing service, which differed from the positive predictive effect of professional capital in the “one-to-one” service [55, 45]. This may be due to the fact that patients who choose healthcare crowdsourcing services are more motivated to seek healthcare services, and thus they are more concerned with whether the doctor’s response addresses the need for consultation, i.e., the information content itself [56]. Rather than relying on external sources of information to make a judgment.

This study provides three theoretical contributions. First, this study expands the relevant research in the field of online healthcare services. Although online healthcare services have received extensive attention from scholars [6, 9, 57], the existing researches mostly focus on the “one-to-one” service and lacks discussion on the medical crowdsourcing service. Second, this study extends the relevant research in the field of crowdsourcing competition to the healthcare field and enriches the existing research on medical crowdsourcing competition. Most of the existing research focus on the role of multi-party crowdsourcing collaboration in public health management, with more emphasis on the cooperative attribute of crowdsourcing [13, 14]. From the perspective of the competitive environment, this paper explores and discusses the factors that influence the success of competition in the medical crowdsourcing service. Third, from the dimensions of service content, most of the existing studies only focus on the quality of response, emotional support and others [34, 58]. This paper further divides the information into four dimensions, namely, conclusive, suggestive, affective and blessed, to better complement and improve the existing relevant studies.

The conclusions of this paper also provide certain enlightenment for all participants of medical crowdsourcing: doctors participating in crowdsourcing competitions, patients who post crowdsourcing tasks and platforms for organizing crowdsourcing competitions. First, our results show that the service content provided by doctors plays an important role in the crowdsourcing competition, especially the conclusive information, affective information and blessed information, which have a positive impact on whether doctors can win in the crowdsourcing competition. Based on the samples in this paper, it can be seen that the mean value of emotional information provided by doctors is close to 0, indicating that doctors’ response has a large room for improvement in this aspect. Therefore, doctors participating in crowdsourcing competitions need to be aware that responses can be structured and organized in addition to focusing on the questions themselves, with conscious inclusion of conclusive, affective and blessed information. On the premise of not affecting the professionalism and accuracy of the response, try to avoid the use of suggestive information, so as to improve their chances of winning.

Second, for patients, setting the reward too high may make doctors less likely to respond. Although higher reward amounts give doctors higher motivation, our results show that higher rewards make crowdsourcing competitions more difficult overall and increase the uncertainty of a doctor’s chances of winning. Therefore, patients should not blindly increase the amount of reward when posting questions to avoid reducing the possibility of doctors participating in the response.

Finally, as the platform manager of the crowdsourcing competition, they can give doctors proper guidance on the response to questions, encourage doctors to include emotional interaction information in the response, and provide certain affective and blessed support to patients, so as to improve their probability of winning the competition. In addition, platform managers should be aware that an overly competitive environment can dynamically affect the probability of a doctor winning. Therefore, when designing the rules of crowdsourcing competitions, the platform should intentionally control the number of doctors participating in the competition and reasonably control the intensity of competition. On the other hand, the difficulty of competition should be balanced, and the entry threshold should be set according to the reward amount of the question, so as to avoid doctors with too big a difference in ability from being in the same competition, and a good competition mechanism should be formed and maintained.

This study has the following limitations. First, the data sources in this paper are limited, and the generalizability of the findings remains to be explored. The Guahao.com platform is a large online medical community in China. Most of the doctors providing services on this platform are from high-level hospitals, and the representativeness of the doctor sample is limited. Moreover, only psychological diseases were selected, which is a limited sample, and the generalizability of the findings needs to be improved. Future studies could select multiple diseases to expand the scope of the study or validate their findings on other platforms. Secondly, it may not be accurate and objective to measure the competition difficulty from the single dimension of reward amount in this study. Future studies may consider taking into account the difficulty of the question to comprehensively judge the competition difficulty. Third, patients can select only 30, 68, and 128 CNY kinds of reward amounts in the crowdsourcing service on Guahao.com, so the role of the reward mechanism may not be fully played. Future studies need to expand the range of reward amounts and explore the role of reward mechanisms in depth. And our study dataset only covered from June through December 2020 and did not have multiple cross-sections in the time series. Future research may consider expanding the time span of the study to more conclusively assess the factors impacting doctors’ wins.

## Data Availability

The data that support the findings of this study are available on request from the corresponding author.
